# TRPV4 Blockade Preserves the Blood–Brain Barrier by Inhibiting Stress Fiber Formation in a Rat Model of Intracerebral Hemorrhage

**DOI:** 10.3389/fnmol.2018.00097

**Published:** 2018-03-27

**Authors:** Hengli Zhao, Kaiyuan Zhang, Rongrui Tang, Hui Meng, Yongjie Zou, Pengfei Wu, Rong Hu, Xin Liu, Hua Feng, Yujie Chen

**Affiliations:** ^1^Department of Neurosurgery, Southwest Hospital, Third Military Medical University, Chongqing, China; ^2^Department of Neurosurgery, Daping Hospital, Third Military Medical University, Chongqing, China

**Keywords:** blood–brain barrier, intracerebral hemorrhage, transient receptor potential vanilloid 4, stress fibers, secondary brain injury

## Abstract

Blood–brain barrier (BBB) disruption and subsequent brain edema play important roles in the secondary neuronal death and neurological dysfunction that are observed following intracerebral hemorrhage (ICH). In previous studies, transient receptor potential vanilloid 4 (TRPV4), a calcium-permeable mechanosensitive channel, was shown to induce cytotoxicity in many types of cells and to play a role in orchestrating barrier functions. In the present study, we explored the role of TRPV4 in ICH-induced brain injury, specifically investigating its effect on BBB disruption. Autologous arterial blood was injected into the basal ganglia of rats to mimic ICH. Adult male Sprague Dawley rats were randomly assigned to sham and experimental groups for studies on the time course of TRPV4 expression after ICH. The selective TRPV4 antagonist HC-067047 and TRPV4 siRNA were administered to evaluate the effects of TRPV4 inhibition. GSK1016790A, a TRPV4 agonist, was administered to naive rats to verify the involvement of TRPV4-induced BBB disruption. A PKC inhibitor, dihydrochloride (H7), and a selective RhoA inhibitor, C3 transferase, were administered to clarify the involvement of the PKCα/RhoA/MLC2 pathway following ICH. Post-ICH assessments including functional tests, brain edema measurements, Evans blue extravasation, western blotting and immunohistochemical assays were performed. TRPV4 inhibition remarkably ameliorated neurological symptoms, brain edema, and neuronal death, as well as BBB disruption, 24–72 h following ICH. Meanwhile, TRPV4 blockade preserved the expression of adherens and tight junction proteins, as well as BBB integrity, by inhibiting stress fiber formation, which might be correlated with the regulation of components of the PKCα/RhoA/MLC2 pathway. Furthermore, adherens and tight junction protein degradation induced by GSK1016790A treatment in naive rats was also related to PKCα/RhoA/MLC2-pathway-mediated stress fiber formation. Based on these findings, therapeutic interventions targeting TRPV4 may represent a novel approach to ameliorate secondary brain injury following ICH.

## Introduction

Spontaneous ICH is a fatal stroke subtype that accounts for ≈15% of all strokes (Keep et al., 2012). According to epidemiological studies, as many as 50% of ICH patients die within the first 48 h, and as few as 20% of survivors return to normal daily living activities (Qureshi, 2011). The development of brain edema is one of the main reasons for the devastating nature of ICH and is believed to be a strong predictor of an unfavorable functional outcome (Gebel et al., 2002; Balami and Buchan, 2012). Although the mechanisms by which edema forms after ICH have not been completely elucidated, the most common type of brain edema, vasogenic edema, is caused by increased BBB permeability. Thus, treatments that attenuate BBB disruption may be a promising strategy for managing ICH.

Transient receptor potential vanilloid 4 (TRPV4), a member of the TRP superfamily, is permeable to Ca^2+^ (Garcia-Elias et al., 2014). TRPV4 is sensitive to various types of stimuli, including mechanical forces, hypoosmotic stimuli, AA metabolites, and exogenous chemical ligands (Shibasaki, 2016). In the central nervous system, TRPV4 is expressed in various cell types, including neurons (Lipski et al., 2006), glial cells (Benfenati et al., 2007; Konno et al., 2012) and vascular endothelial cells (Hatano et al., 2013). TRPV4 plays a pivotal role in central nervous system disorders, such as ischemic stroke (Jie et al., 2015a,b), traumatic brain injury (Lu et al., 2017), and Alzheimer’s disease (Bai and Lipski, 2014). Based on recent evidence, TRPV4 modulates the barrier functions of choroid plexus epithelial cells and capillary endothelial cells in the brain (Brown et al., 2008; Narita et al., 2015). In one study, TRPV4 blockade attenuated BBB damage by inhibiting MMPs in mice with MCAO (Jie et al., 2015b). However, the effects of TRPV4 inhibition on BBB permeability and brain edema following ICH remain unknown.

Intracerebral hemorrhage rapidly triggers robust actin polymerization in endothelial cells, resulting in the formation of force-generating, contractile stress fibers that are mainly composed of F-actin (Burridge and Wittchen, 2013; Manaenko et al., 2016). The contraction of stress fibers induces the formation of intercellular gaps between endothelial cells and the degradation of adherens and tight junction proteins, increasing the permeability of the BBB (Millan et al., 2010). One of the possible mechanisms by which TRPV4 activation contributes to BBB disruption is the formation of stress fibers via activation of the PKCα/RhoA/MLC2 pathway. PKCα regulates endothelial cell permeability downstream of TRPV4 activation in endothelial cells (Adapala et al., 2011). Upon activation, PKCα directly interacts with RhoA and rearranges the cytoskeleton (Huang et al., 2005; Stamatovic et al., 2006). RhoA binds to its downstream effector, Rho-kinase, which then sequentially induces MLC2 phosphorylation and actin stress fiber formation (Srivastava et al., 2013). F-actin accumulation and stress fiber formation critically depend on MLC2 phosphorylation (Shi et al., 2016).

In this study, we aim to investigate the following three hypotheses: (1) TRPV4 inhibition via pharmacological antagonists or a gene silencing approach ameliorates functional deficits and brain edema after experimental ICH in rats; (2) TRPV4 inhibition prevents stress fiber formation, degradation of the adherens and tight junction proteins and subsequent disruption of the BBB after ICH; (3) ICH induces the formation of stress fibers triggered by TRPV4 activation mediated by the PKCα/RhoA/MLC2 pathway.

## Results

### TRPV4 Expression Was Upregulated Following ICH Injury

**FIGURE 1:**
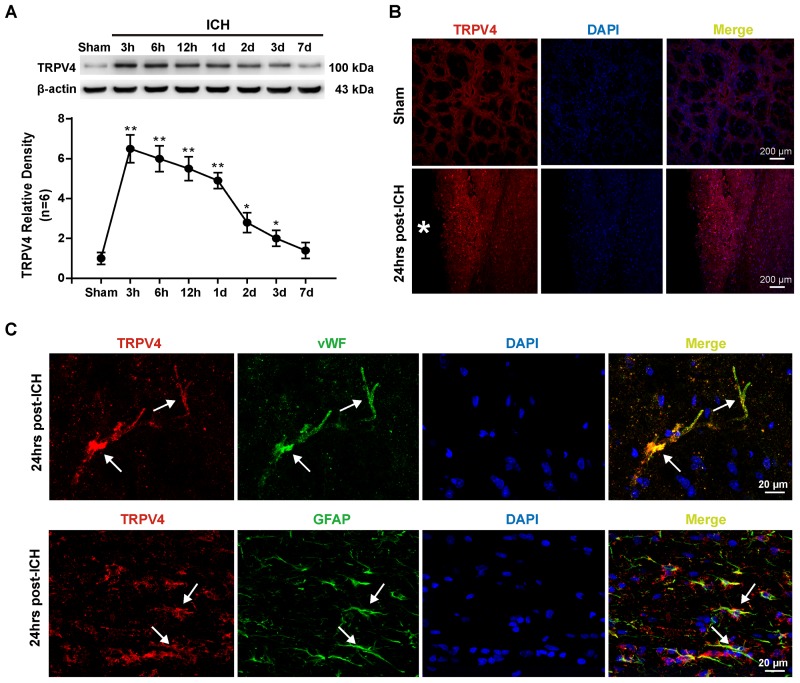
Time course and spatial expression of TRPV4 after ICH. **(A)** Representative bands and quantitative analyses of TRPV4 expression around the lesion sites are shown. Relative densities of each protein were normalized to the sham group. **(B)** Representative images of immunofluorescence staining for TRPV4 (red) in the perihematomal area (white ^∗^) 24 h after ICH are shown. Scale bar: 200 μm. **(C)** Representative images of immunofluorescence staining for TRPV4, vWF (green) and GFAP (green) in the perihematomal area indicated by the white arrow 24 h after ICH are shown. Scale bar: 20 μm, *n* = 6 rats per group. Data are presented as the means ± standard errors of the means. ^∗^*p* **<** 0.05; ^∗∗^*p* < 0.01 compared with the sham group.

The first experiment conducted in this study was to examine the possible alterations in TRPV4 expression in the ipsilateral hemisphere after ICH. Compared with the sham group, TRPV4 levels on western blots were significantly increased 3–24 h after ICH; levels of the TRPV4 protein were fourfold greater than the levels detected in sham rats (*p* < 0.01, **Figure 1A**). Subsequently, TRPV4 expression dramatically decreased and returned close to the level observed in sham rats 7 days after ICH (*p* > 0.05 compared with the sham group, **Figure 1A**). As the profound effect of ICH on TRPV4 levels occurred within 24 h post-ICH, and maximally increased BBB permeability was observed 24 h after ICH, subsequent experiments adopted 24 h as the post-ICH time point.

Immunofluorescence staining also revealed increased TRPV4 expression around the hematoma 24 h after ICH compared with the sham group (*p* < 0.05, **Figure 1B**). Furthermore, double immunofluorescence staining predominantly revealed TRPV4 immunoreactivity on neurovascular structures, including perivascular astrocytes and endothelial cells in the perihematomal area, which were labeled with glial fibrillary acidic protein (GFAP) and von Willebrand factor (vWF), respectively (**Figure 1C**).

### The TRPV4 Antagonist HC-067047 Improved Neurobehavioral Functions and Reduced Brain Edema After ICH

**FIGURE 2:**
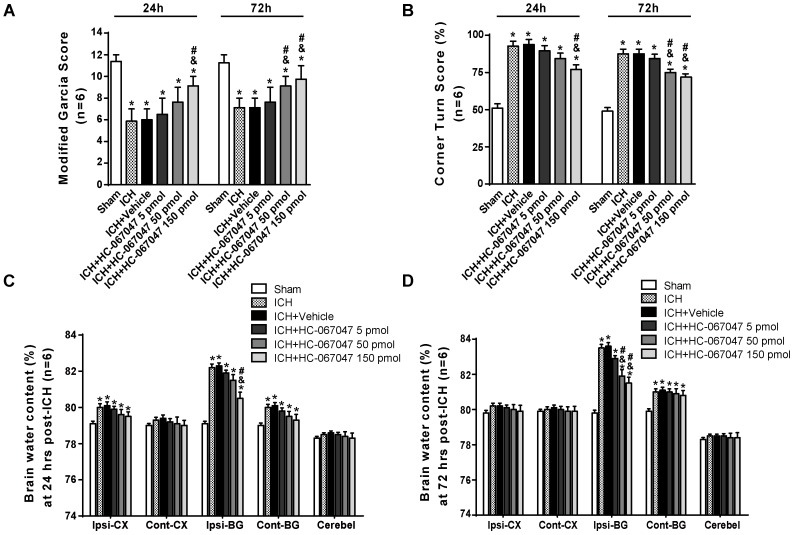
The effects of HC-067047 administration on neurological deficits and brain edema after ICH. **(A)** The results from the modified Garcia test and **(B)** corner turn test obtained from the sham, ICH, vehicle, and HC-067047 treatment groups (5, 50, or 150 pmol per rat) 24 and 72 h after the operation are shown. **(C)** Brain water content assessments at 24 h after the operation in sham, ICH, vehicle, and HC-067047 treatment groups (5, 50, or 150 pmol per rat) are shown. **(D)** Brain water content assessments at 72 h after the operation in sham, ICH, vehicle, and HC-067047 treatment groups (5, 50, or 150 pmol per rat) are shown. Brain specimens were divided into the following four parts: ipsilateral basal ganglia (Ipsi-BG), ipsilateral cortex (Ipsi-CX), contralateral basal ganglia (Cont-BG), and contralateral cortex (Cont-CX). The cerebellum (Cerebel) was used as the internal control, *n* = 6 rats per group. Data are presented as the medians ± 25th–75th percentiles **(A)** or the means ± standard errors of the means **(B–D)**. ^∗^*p* < 0.05 compared with the sham group; ^&^*p* < 0.05 compared with the ICH group; ^#^*p* < 0.05 compared with the vehicle group.

Scores on a modified Garcia test and corner turn test were assessed to examine the effect of the TRPV4 antagonist HC-067047 on neurobehavioral functions on days 1 and 3 after ICH. Compared with the sham group, rats that suffered an ICH injury exhibited significant neurobehavioral deficits both on the modified Garcia test and corner turn test 24 and 72 h after ICH (*p* < 0.05, **Figures 2A,B**). Compared with the untreated ICH group, animals treated with vehicle or 5 pmol of HC-067047 showed no improvement in neurobehavioral deficits at 24 or 72 h after ICH (*p* > 0.05), but the 50- and 150-pmol HC-067047 treatments significantly ameliorated neurobehavioral outcomes at 72 h after ICH (*p* < 0.05, **Figure 2B**), whereas only the group treated with 150 pmol showed any benefit at 24 h after ICH compared with the ICH group and vehicle group (*p* < 0.05, **Figure 2A**).

At 24 h after ICH, the brain water content in the ipsilateral cortex and basal ganglia, as well as in the contralateral basal ganglia, was significantly increased compared with that in the sham group (*p* < 0.05). In the ipsilateral basal ganglia and contralateral basal ganglia, the brain water content was increased at 72 h post-ICH (*p* < 0.05, **Figures 2C,D**). Compared with a lack of treatment following ICH, treatment with vehicle or 5 pmol of HC-067047 did not reduce the percent water content in the ipsilateral basal ganglia at 24 or 72 h after ICH (*p* > 0.05, **Figures 2C,D**). However, the 50- and 150-pmol HC-067047 treatments significantly reduced brain water content in the ipsilateral basal ganglia at 72 h after ICH (*p* < 0.05, **Figure 2D**), but only the 150-pmol treatment reduced the brain water content in the ipsilateral basal ganglia at 24 h post-ICH compared with the ICH group and vehicle group (*p* < 0.05, **Figure 2C**). The HC-067047 treatment did not reduce brain water content in the ipsilateral cortex or contralateral basal ganglia at 24 h post-ICH or in the contralateral basal ganglia at 72 h post-ICH (*p* > 0.05, **Figures 2C,D**).

### The TRPV4 Antagonist HC-067047 Attenuated ICH-Induced Neuronal Damage and Disruption of the BBB Without Affecting Hematoma Volume

**FIGURE 3:**
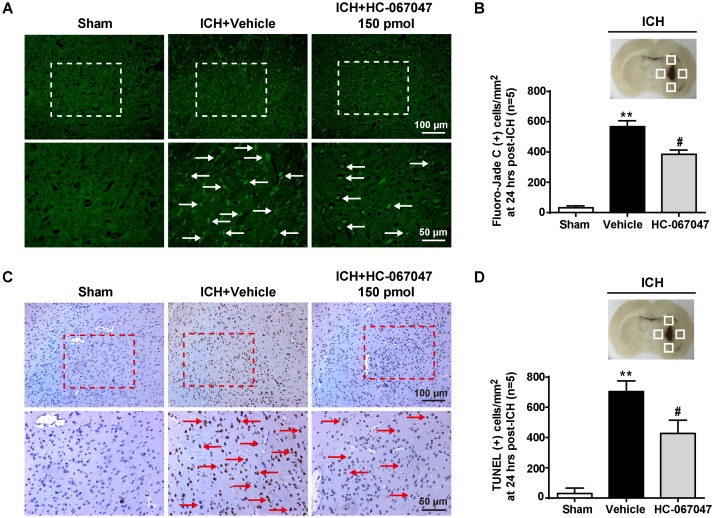
The effects of HC-067047 administration on neuronal damage after ICH. **(A)** Representative images of F-JC staining and **(B)** quantitative analyses of F-JC-positive neurons in sham, vehicle, and HC-067047 treatment groups (150 pmol per rat) 24 h after the operation are shown. Scale bar: 100 or 50 μm. **(C)** Representative images of TUNEL staining and **(D)** quantitative analyses of TUNEL-positive cells in the sham, vehicle, and HC-067047 treatment groups (150 pmol per rat) 24 h after the operation are shown. Scale bar: 100 or 50 μm. The schematic diagram shows the four areas (white squares) used for counting F-JC positive cells (white arrows in panel **A**) and TUNEL positive cells (red arrows in panel **C**) in the perihematomal region, *n* = 5 rats per group. Data are presented as the means ± standard errors of the means. ^∗∗^*p* < 0.01 compared with the sham group; ^#^*p* < 0.05 compared with the vehicle group.

We employed F-JC staining to evaluate the neuronal damage around the hematoma. ICH induced a significant increase in the number of F-JC positive neurons (*p* < 0.01 compared with the sham group), and the 150-pmol HC-067047 treatment decreased the level of neuronal damage in the perihematomal area compared with the level in the vehicle group at 24 h after ICH (*p* < 0.05, **Figures 3A,B**). In addition, compared with the sham group, the ICH + vehicle group exhibited an increased number of TUNEL-positive cells in the perihematomal area at 24 h after ICH (*p* < 0.01), indicating a substantial increase in apoptosis. Remarkably, the 150-pmol HC-067047 treatment significantly reduced the number of TUNEL-positive cells and effectively prevented apoptotic cell death (*p* < 0.05, **Figures 3C,D**).

**FIGURE 4:**
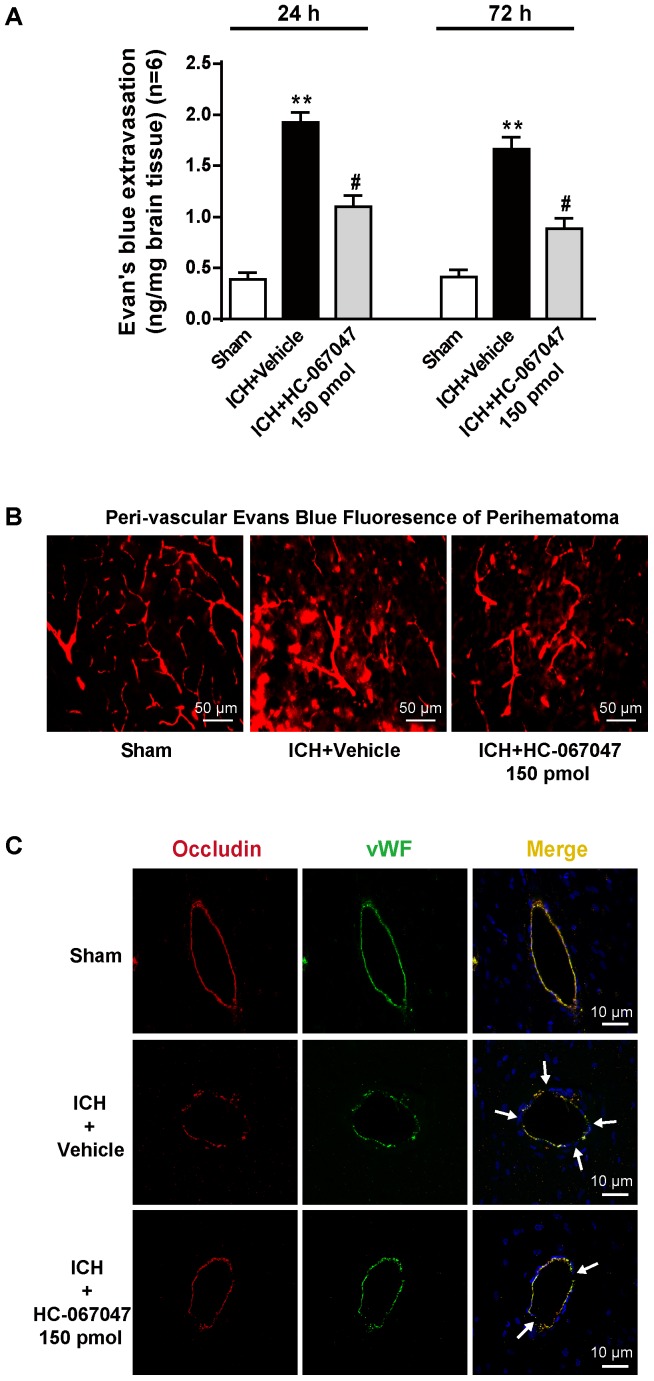
The effects of HC-067047 administration on BBB integrity after ICH. **(A)** The Evans blue extravasation evaluation was performed in the sham, vehicle, and HC-067047 treatment groups (150 pmol per rat) 24 and 72 h after the operation. **(B)** Representative images of Evans blue fluorescence observed around the lesion sites in the sham, vehicle, and HC-067047 treatment groups (150 pmol per rat) 24 h after the operations are shown. Scale bar: 50 μm. **(C)** Representative images of immunofluorescence staining for Occludin and vWF around the lesion sites in the sham, vehicle, and HC-067047 treatment groups (150 pmol per rat) 24 h after the operation are shown. The arrow indicates the breakdown of the continuous endothelial cell layer. Scale bar: 10 μm, *n* = 6 rats per group. Data are presented as the means ± standard errors of the means. ^∗∗^*p* < 0.01 compared with the sham group; ^#^*p* < 0.05 compared with the vehicle group.

In the Evans blue extravasation experiment, a greater amount of extravasated dye in the ipsilateral brain parenchyma was exhibited by the ICH + vehicle group at 24 and 72 h after ICH than the sham group (*p* < 0.01), but the 150-pmol HC-067047 treatment significantly reduced dye leakage (*p* < 0.05 compared with the vehicle group, **Figure 4A**). In addition, Evans blue fluorescence revealed a greater amount of extravasated Evans blue dye around arterioles in the ICH + vehicle group than in the sham group, and the 150-pmol HC-067047 treatment reduced perivascular Evans blue dye leakage (**Figure 4B**). Furthermore, immunofluorescence staining showed that the structures of continuous endothelial cells (vWF) and Occludin observed in the sham group were disrupted in the ICH + vehicle group, whereas the 150-pmol HC-067047 treatment effectively reduced the damage (**Figure 4C**).

The hematoma volume in the ICH group was observed using T2^∗^-weighted MRI (**Supplementary Figures S1A,B**). The ipsilateral hemisphere was damaged 24 h after ICH, but no significant difference in hematoma size was observed between the ICH + vehicle group and the ICH + 150-pmol HC-067047 group (*p* > 0.05). Thus, our ICH rat models were reproducible and consistent, and the neuroprotective effects of HC-067047 were not mediated by reducing the expansion of the hematoma after ICH.

### The TRPV4 Antagonist HC-067047 Attenuated Both the ICH-Induced Degradation of Adherens and Tight Junction Proteins and Activation of the PKCα/RhoA/MLC2 Pathway

**FIGURE 5:**
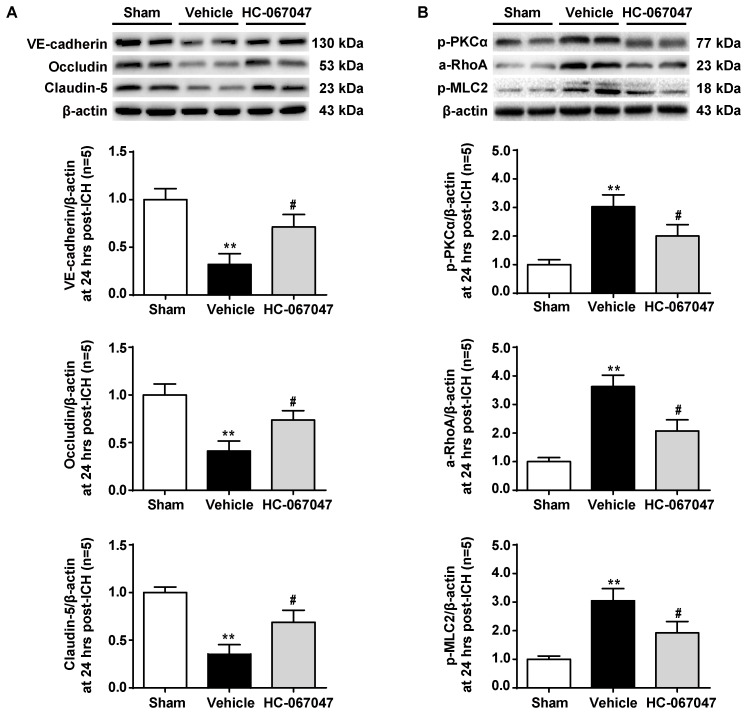
The effects of HC-067047 administration on the expression of adherens/tight junction proteins and activation of the PKCα/RhoA/MLC2 pathway after ICH. **(A)** Representative bands and quantitative analysis of the expression levels of VE-Cadherin, Occludin, and Claudin-5 24 h following the operation in the sham, vehicle, and HC-067047 treatment groups (150 pmol per rat) are shown. **(B)** Representative bands and quantitative analysis of the activation of the PKCα/RhoA/MLC2 pathway 24 h following the operation in the sham, vehicle, and HC-067047 treatment groups (150 pmol per rat) are shown, *n* = 5 rats per group. Data are presented as the means ± standard errors of the means. ^∗∗^*p* < 0.01 compared with the sham group; ^#^*p* < 0.05 compared with the vehicle group.

As BBB permeability is regulated by the adherens and tight junctions, we first tested the effects of HC-067047 on the levels of VE-cadherin, Occludin, and Claudin-5 proteins 24 h after ICH using western blotting (**Figure 5A**). Vehicle-treated ICH rats showed significantly decreased expression of adherens and tight junction proteins (*p* < 0.01 compared with the sham group). However, after the administration of HC-067047, the levels of adherens and tight junction proteins were increased (*p* < 0.05 compared with the vehicle group).

The potential downstream effectors of TRPV4 signaling were examined to investigate the molecular mechanisms underlying the protective effects of HC-067047 and the expression of the PKCα/RhoA/MLC2 pathway (**Figure 5B**). Levels of phosphorylated PKCα were significantly increased 24 h after ICH (*p* < 0.01 compared with the sham group), but this effect was reduced by HC-067047 administration (*p* < 0.05 compared with the vehicle group). In addition, HC-067047 also attenuated increased RhoA activity and MLC2 phosphorylation induced by ICH injury.

### TRPV4 Knock-Down Preserved the BBB and Attenuated Neurological Deficits After ICH

**FIGURE 6:**
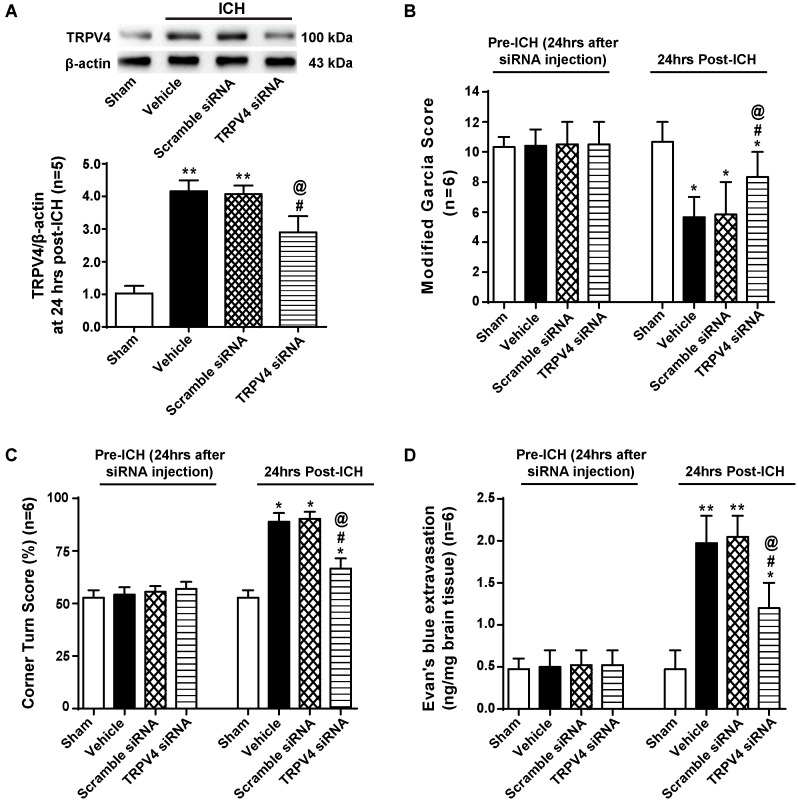
The effects of TRPV4 siRNA on neurological deficits and BBB integrity after ICH. **(A)** Representative bands and quantitative analysis of the inhibitory effect of the TRPV4 siRNA are shown. Relative densities of each protein were normalized to the sham group, *n* = 5 rats per group. **(B)** A modified Garcia test and **(C)** corner Turn test were performed on the sham, vehicle, scrambled siRNA and TRPV4 siRNA groups 24 h after the operation. **(D)** The Evans blue extravasation evaluation was performed on the sham, vehicle, scrambled siRNA, and TRPV4 siRNA groups 24 h after the operation, *n* = 6 rats per group. Data are presented as the means ± standard errors of the means. ^∗∗^*p* < 0.01 and ^∗^*p* < 0.05 compared with the sham group; ^#^*p* < 0.05 compared with the vehicle group; ^@^*p* < 0.05 compared with the scrambled siRNA group.

To confirm our results, we used TRPV4 siRNA, which is a more specific method for targeting this receptor. TRPV4 expression was measured by western blotting, and compared with the sham group, TRPV4 expression in the ipsilateral hemisphere was significantly increased at 24 h after ICH (*p* < 0.01, **Figure 6A**). No differences were observed in the scrambled siRNA pretreatment group or vehicle group (*p* > 0.05), but compared with the vehicle group and the scrambled siRNA group, the TRPV4 siRNA pretreatment significantly inhibited the ICH-induced increase in TRPV4 expression (*p* < 0.05, **Figure 6A**).

Neurobehavioral functions and BBB permeability were evaluated 24 h following ICH (**Figures 6B–D**). No neurological impairments were observed following the siRNA injection, but Evans blue extravasation was detected (*p* > 0.05). However, both vehicle- and scrambled siRNA-pretreated ICH rats developed significant neurological deficits and BBB disruption 24 h after ICH compared to sham rats (*p* < 0.05 and *p* < 0.01, respectively), and no significant differences were observed between the vehicle group and scrambled siRNA group (*p* > 0.05). TRPV4 siRNA-pretreated rats performed better on both the modified Garcia test and corner turn test compared with the vehicle group and scrambled siRNA group (*p* < 0.05). Similarly, TRPV4 siRNA-pretreated rats showed less Evans blue dye extravasation compared to the vehicle group and scrambled siRNA group (*p* < 0.05).

### TRPV4 Knock-Down Attenuated Both the ICH-Induced Degradation of Adherens and Tight Junction Proteins and Activation of the PKCα/RhoA/MLC2 Pathway

**FIGURE 7:**
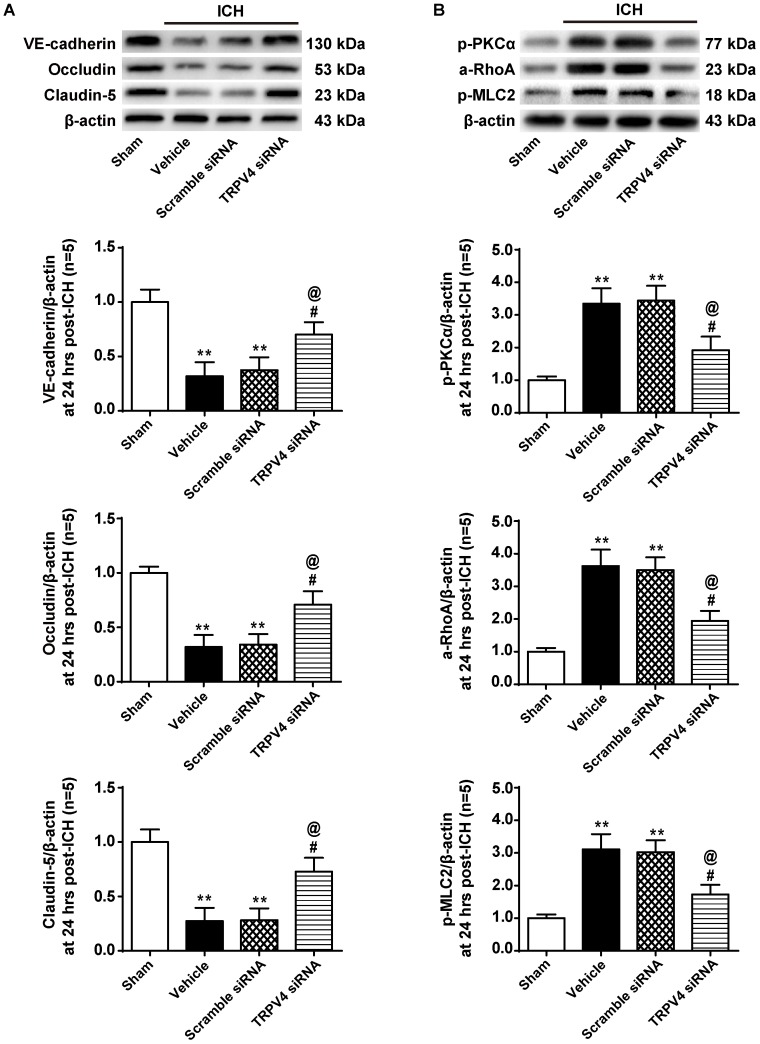
The effects of TRPV4 siRNA on the expression of adherens/tight junction proteins and activation of the PKCα/RhoA/MLC2 pathway after ICH. **(A)** Representative bands and quantitative analysis of the expression levels of VE-Cadherin, Occludin, and Claudin-5 in the sham, vehicle, scramble siRNA, and TRPV4 siRNA groups 24 h after the operation are shown. **(B)** Representative bands and quantitative analysis of the activation of the PKCα/RhoA/MLC2 pathway in the sham, vehicle, scramble siRNA, and TRPV4 siRNA groups 24 h after the operation are shown, *n* = 5 rats per group. Data are presented as the means ± standard errors of the means. ^∗∗^*p* < 0.01 compared with the sham group; ^#^*p* < 0.05 compared with the vehicle group; ^@^*p* < 0.05 compared with the scrambled siRNA group.

Correspondingly, compared with the vehicle group, the TRPV4 siRNA pretreatment significantly increased the expression of VE-cadherin, Occludin, and Claudin-5, whereas the scrambled siRNA did not exert this effect (*p* < 0.05 and *p* > 0.05, respectively, **Figure 7A**). Moreover, the TRPV4 siRNA pretreatment also significantly reduced the ICH-induced activation of the PKCα/RhoA/MLC2 pathway compared to the vehicle group and scrambled siRNA group (*p* < 0.05, **Figure 7B**).

### The TRPV4 Agonist GSK1016790A Induced the Degradation of Adherens and Tight Junction Proteins and Activation of the PKCα/RhoA/MLC2 Pathway in Naive Animals

**FIGURE 8:**
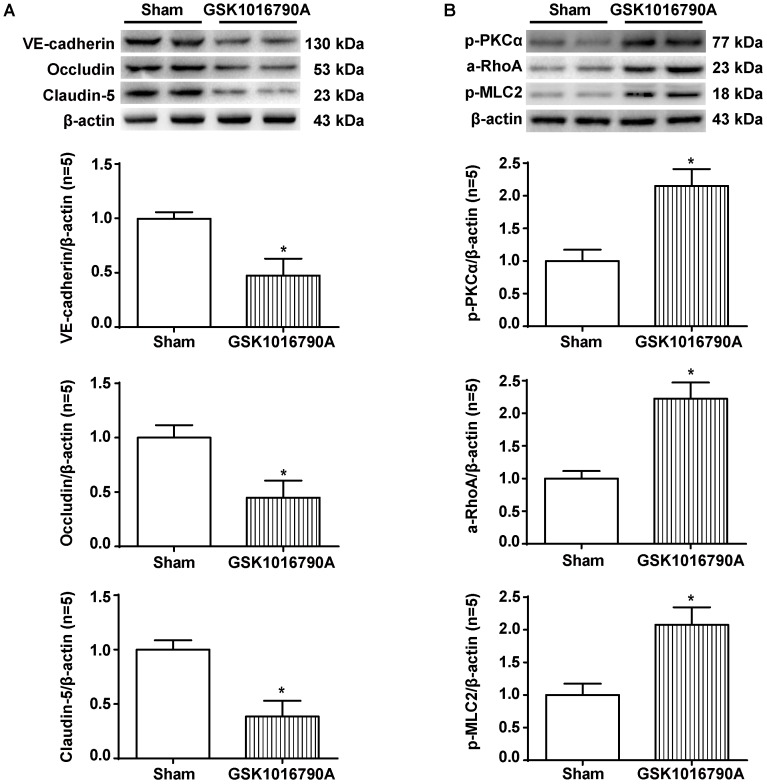
The effects of GSK1016790A on the expression of adherens/tight junction proteins and activation of the PKCα/RhoA/MLC2 pathway after ICH. **(A)** Representative bands and quantitative analysis of the expression levels of VE-Cadherin, Occludin, and Claudin-5 in the sham and GSK1016790A groups 24 h after the operation are shown. **(B)** Representative bands and quantitative analysis of the activation of the PKCα/RhoA/MLC2 pathway in the sham and GSK1016790A groups 24 h after the operation are shown, *n* = 5 rats per group. Data are presented as the means ± standard errors of the means. ^∗^*p* < 0.05 compared with the sham group.

Twenty-four hours after the injection of the TRPV4 agonist GSK1016790A into the brains of naive rats, we observed significant reductions in the levels of VE-cadherin, Occludin, and Claudin-5 proteins compared to the sham group (*p* < 0.05, **Figure 8A**). In addition, GSK1016790A alone activated the PKCα/RhoA/MLC2 pathway (*p* < 0.05, compared with the sham group, **Figure 8B**).

### ICH and GSK1016790A Induced the Formation of Stress Fibers, Whereas HC-067047 and TRPV4 Knock-Down Decreased Stress Fiber Formation After ICH

**FIGURE 9:**
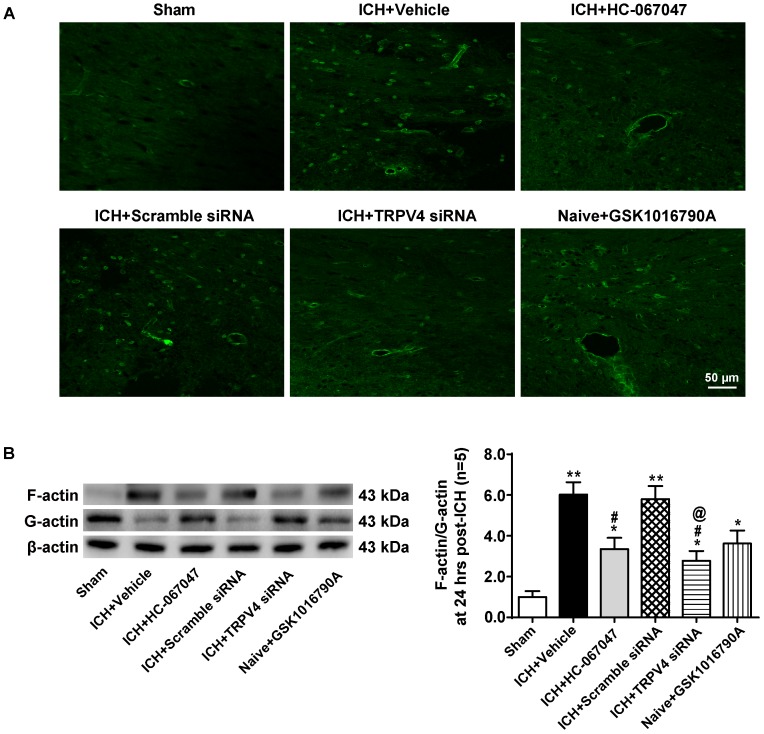
The effects of HC-067047, TRPV4 siRNA, and GSK1016790A on the formation of F-actin stress fibers after ICH. **(A)** Representative images of phalloidin fluorescence around the lesion sites in the sham, vehicle, HC-067047, TRPV4 siRNA, and GSK1016790A groups captured 24 h after the operation. Scale bar: 50 μm. **(B)** Representative bands and quantitative analysis of the expression levels of polymerized F-actin, soluble G-actin, and total β-actin in the sham, vehicle, HC-067047, TRPV4 siRNA, and GSK1016790A groups 24 h after the operation are shown. The ratio of F-actin to G-actin was quantified as a measure of stress fiber formation, *n* = 5 rats per group. Data are presented as the means ± standard errors of the means. ^∗∗^*p* < 0.01 compared with the sham group; ^∗^*p* < 0.05 compared with the sham group; ^#^*p* < 0.05 compared with the vehicle group; ^@^*p* < 0.05 compared with the scrambled siRNA.

Phalloidin fluorescence (**Figure 9A**) revealed a dramatic increase in the number of phalloidin-positive cells 24 h after ICH compared to the sham group (*p* < 0.01), indicating the formation of F-actin^+^ stress fibers. The morphology of phalloidin-positive cells was similar to that of neurovascular structures, such as perivascular astrocytes and endothelial cells. TRPV4 inhibition via a pharmacological antagonist or via the gene silencing approach decreased stress fiber formation considerably (*p* < 0.05 compared with the vehicle group), whereas the scrambled siRNA did not affect this parameter (*p* > 0.05 compared with the vehicle group). Notably, compared to the sham group, the GSK1016790A treatment directly increased the number of stress fibers in the brains of naive rats (*p* < 0.05).

Levels of total β-actin, polymerized F-actin and soluble G-actin were examined by western blotting 24 h after the operation (**Figure 9B**). The ratio of F-actin to G-actin was quantified as a measure of stress fiber formation. Consistent with the findings described above, compared with the sham group, ICH significantly increased the F-actin to G-actin ratio (*p* < 0.05), and the TRPV4 blockade by HC-067047 and the TRPV4 siRNA reduced this ratio (*p* < 0.05 compared with the vehicle group). Moreover, compared to the sham group, GSK1016790A administration directly increased the F-actin to G-actin ratio in the brains of naive rats (*p* < 0.05).

### Specific Inhibition of the PKCα/RhoA/MLC2 Pathway Attenuated BBB Permeability After ICH

The PKC inhibitor, H7, and RhoA inhibitor, C3 transferase, were administered after the operation to evaluate their effects on the expression of downstream molecules and Evans blue extravasation as well as to clarify the specific influence of the PKCα/RhoA/MLC2 pathway on BBB disruption after ICH. H7 significantly attenuated the increase in RhoA activity and MLC2 phosphorylation induced by ICH injury (*p* < 0.01 compared with the vehicle group, **Supplementary Figure S2A**), and the administration of C3 transferase also remarkably diminished ICH-induced MLC2 phosphorylation (*p* < 0.01 compared with the vehicle group, **Supplementary Figure S2B**). In addition, both H7 and C3 transferase notably reduced the leakage of Evans blue dye and preserved the BBB after ICH (*p* < 0.05 compared with the vehicle group, **Supplementary Figure S2C**).

## Discussion

Intracerebral hemorrhage is a devastating clinical event without effective therapies. After the sudden rupture of cerebral blood vessels, a hematoma rapidly forms in the brain and compresses surrounding brain tissues, leading to the “mass effect” (Qureshi et al., 2009). Hematoma-induced mechanical stress and blood metabolites trigger a series of life-threatening events, leading to the accumulation of cerebral edema, progression of neurobehavioral deficits, and possibly death (Strbian et al., 2008). BBB disruption is one of the most important pathophysiological changes in hematoma-induced injury after ICH and contributes to vasogenic brain edema formation, which leads to a poor prognosis (Yang and Rosenberg, 2011). In the present study, we investigated the roles of TRPV4 and its ability to orchestrate BBB disruption following an ICH injury. In our findings, a relatively high dosage (150 pmol per rat) of the TRPV4 antagonist HC-067047 alleviated the neurological deficits and Evans blue leakage observed after the experimental induction of ICH, as did TRPV4 siRNA; this effect of TRPV4 blockade was probably associated with the inhibition of vasogenic edema. Thus, TRPV4 blockade may be a novel strategy for the treatment of patients with ICH.

The BBB is composed of endothelial cells, astrocyte end-feet, pericytes, and a thick basement membrane that serves as a dynamic semipermeable barrier separating the peripheral circulation and the central nervous system (Abbott et al., 2010). The permeability of the BBB is regulated by adherens and tight junctions. Adherens junctions provide mechanical strength to cell-cell adhesions via VE-cadherin, which is a transmembrane protein that is anchored in the cytoskeleton (Wojciak-Stothard et al., 2001), and tight junctions mediate the gate functions of the BBB (Schlunk and Greenberg, 2015). The formation of tight junctions depends on the expression of high levels of Occludin and Claudin-5 and intermediates in intracellular signaling pathways that control the phosphorylation of junctional proteins (Morita et al., 1999; Rubin and Staddon, 1999). Actin in the endothelium may be linked to Occludin/Claudins through multiple accessory proteins, such as ZO proteins, and may participate in regulating tight junctions (Fanning et al., 1998). Therefore, dynamic interactions between the cytoskeleton and junctional proteins are important for BBB maintenance. According to emerging evidence, the endothelial actin cytoskeleton shifts from monomeric G-actin into a F-actin under pathophysiological conditions, including ICH, leading to stress fiber formation (Adyshev et al., 2013; Manaenko et al., 2016; Shi et al., 2016). The contraction of stress fibers results in the formation of intercellular gaps between endothelial cells and the degradation of adherens and tight junction proteins, thereby increasing the permeability of the BBB (Millan et al., 2010).

RhoA is a small guanosine triphosphate-binding protein known to regulate the formation of focal adhesions and stress fibers in response to growth factors (Fujita and Yamashita, 2014). Upon activation, RhoA binds to its downstream effector Rho-kinase, which then sequentially induces MLC2 phosphorylation and actin stress fiber formation, leading to cytoskeletal reorganization and increased BBB permeability (Srivastava et al., 2013). Based on the results from our laboratory and other groups, increased RhoA activity contributes to the ICH-induced early disruption of the BBB, and RhoA inhibition attenuates stress fiber formation and the degradation of adherens and tight junction proteins, thereby preserving the BBB after ICH (Huang et al., 2012; Manaenko et al., 2016; Zhao et al., 2016). In the present study, TRPV4 inhibition alleviated increases in RhoA activity, MLC2 phosphorylation, and stress fiber formation after ICH, implying that mechanisms regulating RhoA/p-MLC2 may mediate the preservation of BBB integrity induced by TRPV4 inhibition.

PKC isozymes are serine-threonine kinases that phosphorylate multiple proteins, which in turn regulate intracellular signaling. A large influx of intracellular Ca^2+^ often leads to the activation of members of the PKC family, particularly PKCα, which plays a critical role in regulating endothelial cell permeability (Sandoval et al., 2001; Holinstat et al., 2003). In one study, PKCα expression peaked 6 h after autologous blood-induced ICH, whereas treatment with a PKCα inhibitor profoundly attenuated ICH-induced BBB breakdown (Cui et al., 2011). Upon activation, PKCα directly interacts with RhoA and then induces cytoskeletal reorganization (Huang et al., 2005; Stamatovic et al., 2006). Thrombin was recently shown to trigger a visible increase in PKCα phosphorylation and significant translocation of PKCα to the membrane, leading to increased RhoA activity, MLC2 phosphorylation and stress fiber formation *in vitro* (Sandoval et al., 2001; Wang et al., 2016). Similarly, pharmacological inhibition of PKCα reduced the formation of focal adhesions and stress fibers, as well as RhoA activity in astrocytes (Avalos et al., 2009). In the present study, TRPV4 suppression reduced PKCα activation, stress fiber formation and the degradation of adherens and tight junction proteins after ICH, implying that PKCα is involved in the mechanism by which TRPV4 inhibition protects the BBB.

The most immediate impact of the “mass effect” is the substantial mechanical stress that it causes on the surrounding brain tissues, and the presence of a larger “mass effect” worsens the prognosis of patients with ICH (Keep et al., 2012). As the results of STICH I and STICH II confirmed that early surgical clearance of hematomas does not improve the prognosis of patients with ICH (Mendelow et al., 2005, 2013), direct removal of mechanical stress caused by the hematoma is not effective. Therefore, attention should be paid to strategies aimed at alleviating mechanical stress-induced damage. Recently, TRPV4-induced intracellular Ca^2+^ overload was shown to mediate mechanical stress-induced damage (O’Neil and Heller, 2005) and participate in pathophysiological processes, such as vasoconstriction, edema, and apoptosis, eventually leading to pathological changes, such as pulmonary vascular endothelial injury (Jian et al., 2008) and myocardial ischemia (Dong et al., 2017). TRPV4 is widely expressed in the central nervous system and functions not only in neurons and glial cells but also in endothelial cells and smooth muscle cells in cerebral arteries (Nilius and Voets, 2013; Nilius and Szallasi, 2014). In the present study, TRPV4 expression was significantly upregulated as early as 3 h following autologous blood-induced ICH injury, and double immunofluorescence staining mainly revealed TRPV4 immunoreactivity on the neurovascular structures, including perivascular astrocytes and endothelial cells in the perihematomal area. Considering the high sensitivity of TRPV4 to mechanical stress, we propose that the rapid formation of a hematoma crushes the surrounding cells and dramatically activates the Ca^2+^-permeable TRPV4 channel; TRPV4 activation induces Ca^2+^ entry and activates the Ca^2+^-dependent PKCα/RhoA/MLC2 pathway, thereby leading to the formation of stress fibers and disruption of the BBB during ICH. However, as for mechanical force, brain-edema-induced osmotic stress (Vriens et al., 2004) and shear stress caused by metabolism-related changes in cerebral blood flow (Baratchi et al., 2016) might also contribute to the activation of TRPV4 after ICH. Nevertheless, brain edema and the metabolism-related change in cerebral blood flow after ICH occur gradually rather than immediately; therefore, we speculated that the significant upregulation of TRPV4 at the hyperacute stage was related primarily to the mechanical stress of hematoma. Admittedly, the specific molecules that regulate the expression of TRPV4 after ICH remain unknown and require further exploration.

We also investigated whether the injection of the TRPV4 selective agonist GSK1016790A into the brains of naive animals would activate the proposed pathway and induce the degradation of BBB proteins to verify the involvement of TRPV4-induced BBB disruption. An intracerebroventricular injection of GSK1016790A dose-dependently induced apoptosis in mouse hippocampi and exerted neurotoxic effects (Jie et al., 2016). Here, GSK1016790A administration increased the number of stress fibers, accompanied by PKCα/RhoA/MLC2 activation and the degradation of adherens and tight junction proteins. Furthermore, the involvement of the PKCα/RhoA/MLC2 pathway in ICH-induced BBB disruption was also confirmed in the present study by using the PKC inhibitor H7 and the RhoA inhibitor C3 transferase.

Several potential weaknesses of our study should be mentioned. First, as we mentioned above, in addition to hematoma-induced mechanical stress, cell-swelling-induced osmotic alterations and metabolites of AA released in response to membrane phospholipid degradation may also contribute to TRPV4 activation after ICH; thus, further studies should be performed to clarify these issues. Second, TRPV4 and F-actin may co-exist in distinct membrane structures (Becker et al., 2009; Shin et al., 2012). Therefore, we cannot exclude the possibility that other mechanisms also play a role in TRPV4-mediated stress fiber formation. Moreover, we only evaluated neuroprotective effects of TRPV4 suppression within 3 days after ICH in this study. The long-term outcomes of TRPV4 inhibition after ICH should be assessed.

**FIGURE 10:**
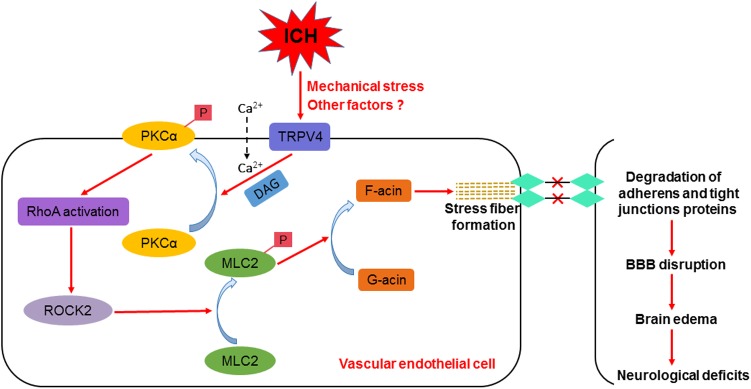
Schematic of the effect of TRPV4 on BBB integrity after ICH.

In summary, we are the first group to show that TRPV4 at least partially contributes to ICH-induced stress fiber formation, BBB impairments, and impaired neurological functions via the PKCα/RhoA/MLC2 pathway (**Figure 10**). Therefore, treatments targeting TRPV4 signaling may pave the way for a new therapeutic strategy for patients with ICH, and this topic warrants further research.

## Materials and Methods

### Rat ICH Model

Two hundred forty-one male Sprague Dawley rats (300–320 g) were provided by the Experimental Animal Center of the Third Military Medical University (Chongqing, China). The rats were housed under specific-pathogen-free conditions and had free access to food and water. All experimental procedures were approved by the Ethics Committee of Southwest Hospital and were performed in accordance with the guidelines of the National Institutes of Health Guide for the Care and Use of Laboratory Animals. The rat ICH model was established as described in our previous study (Yang et al., 2015). Briefly, animals were anesthetized with an intraperitoneal injection of sodium pentobarbital (50 mg/kg body weight) and placed on a stereotaxic apparatus (Xi’an Northwest Optical Instrument Factory, Xi’an, China). A whole (1 mm) was drilled in the skull, and a needle was inserted into the right basal ganglia under stereotactic guidance (coordinates: 0.2 mm anterior, 5.5 mm ventral, and 3.5-mm lateral to the midline). Then, 100 μL of autologous blood, taken from the right femoral artery, was infused slowly (5 μL/min) with a microinfusion pump. After the infusion was complete, the needle was left in place for an additional 10 min to prevent backflow before withdrawal. Control animals received a sham operation with needle insertion only. Bone wax was placed around the burr hole, and the skin incision was closed with sutures in both the experimental and control groups. All the procedures were conducted under aseptic conditions to avoid infection.

### Intracerebroventricular Infusion

Intracerebroventricular drug injections were performed as described in our previous study (Zhao et al., 2016). Briefly, rats were anesthetized with an intraperitoneal injection of pentobarbital (50 mg/kg) and then fixed onto a stereotaxic head apparatus. A 26-gauge stainless steel needle attached to a 10-μL Hamilton syringe (Microliter 701; Hamilton Company, Reno, NV, United States) was inserted into the left lateral ventricle through a cranial burr hole (0.3 mm posterior, 1.0 mm lateral, and 2.5 mm ventral to the bregma) at a rate of 0.5 μL/min. The needle was left in place for an additional 15 min after the infusion, and then the incision was closed with sutures. Repeated intracerebroventricular injections of drugs were performed by first implanting a 23-gauge stainless steel guide cannula (Plastics One, Roanoke, VA, United States) into the left lateral ventricle that was anchored to the skull with four stainless steel screws and dental cement.

### Drug Administration

Various concentrations of the selective TRPV4 antagonist HC-067047 (5, 50, or 150 pmol per rat; Sigma-Aldrich, St. Louis, MO, United States) were intracerebroventricularly injected into rats after ICH. For the 72-h study, HC-067047 was administered three times at 0.5, 24, and 48 h after ICH. A single intracerebroventricular injection of the selective TRPV4 agonist GSK1016790A (Sigma-Aldrich, St. Louis, MO, United States) was administered to naive rats at a dosage of 50 pmol per rat. The above antagonists or agonists were first dissolved in DMSO and then in 0.9% saline to a final volume of 5 μL and a final DMSO concentration of 1%. The PKC inhibitor H7 (Tocris Company, United Kingdom) was dissolved in saline (0.9% NaCl) and injected intraperitoneally into the rat at a dosage of 1 mg/kg body weight (Cui et al., 2011). The RhoA-selective inhibitor C3 transferase (40 ng/rat, Cytoskeleton, Denver, CO, United States) was dissolved in saline (0.9% NaCl) and independently administered to the hematoma 30 min after ICH (Fu et al., 2014; Zhao et al., 2016).

Three unique 27-mer TRPV4 siRNA duplexes (OriGene, Rockville, MD, United States) were mixed to enhance the gene silencing efficacy. A scrambled siRNA (OriGene, Rockville, MD, United States) was used as a control. Five hundred picomoles of TRPV4 siRNA or scrambled siRNA in 5 μL was diluted with Lipofectamine 2000 (Life Technologies, Shanghai, China) according to the manufacturer’s protocol and infused intracerebroventricularly 48 h before ICH modeling.

### Functional Behavioral Test

A 12-point scoring system named the modified Garcia test was conducted in a blinded fashion as described in our previous report (Zhao et al., 2016). Behavioral deficits were evaluated 24 and 72 h after ICH. Briefly, this test consisted of four individual tests of side stroke, vibrissae touch, limb symmetry, and lateral turning. A score ranging from 0 (worst performance) to 3 (best performance) was given for each sub-test, and a total Garcia score was calculated as the sum of all subtest scores.

The corner turn test was also conducted 24 and 72 h after ICH to assess the rats’ motor function. Briefly, rats were allowed to walk into a 30-degree corner. When exiting the corner, rats could either turn left or right, and this choice was recorded. Trials were repeated 10 times with 30-s intervals, and the percentage of right turns was calculated.

### Analysis of Brain Water Content

Brain water content was examined at 24 and 72 h after ICH using our previously described method (Zhao et al., 2016). Briefly, rats were euthanized under deep anesthesia, and their brains were immediately removed. Brain specimens were quickly divided into the following five parts: ipsilateral and contralateral cortices, ipsilateral and contralateral basal ganglia, and cerebellum. Each part was weighed on an electric analytical balance to obtain the wet weight and then dried at 100°C for 72 h to obtain the dry weight. The brain water content was calculated as a percentage using the following formula:

(wet weight-dry weight)/wet weight×100%.

### Immunofluorescence Staining

Twenty-four hours after ICH, rats were transcardially perfused with phosphate-buffered saline (PBS, pH 7.4) followed by 10% paraformaldehyde under deep anesthesia. Brains were removed and fixed with formalin at 4°C overnight. Samples were then dehydrated with 30% sucrose in phosphate-buffered saline (PBS, pH 7.4), and frozen sections (10 μm thick) were obtained using a cryostat (CM3050S; Leica Microsystems, Buffalo Grove, IL, United States). Immunofluorescence staining was performed as previously described. Briefly, sections were incubated with 5% bovine serum albumin for 30 min and incubated with the following primary antibodies overnight at 4°C: rabbit polyclonal anti-TRPV4 antibody (1:200, Alomone Labs, Jerusalem, Israel); goat polyclonal anti-vWF antibody (1:200, Invitrogen, Grand Island, NY, United States); goat polyclonal anti-GFAP antibody (1:500, Abcam, Cambridge, MA, United States); and rabbit polyclonal anti-Occludin antibody (1:125, Invitrogen, Grand Island, NY, United States). The specimens were incubated with appropriate secondary antibodies for 3 h at 37°C and then counterstained with DAPI for 5 min. F-actin stress fibers in each specimen were stained with an Alexa Fluor-phalloidin kit (100 nM, Cytoskeleton, Inc.), as previously described (Manaenko et al., 2016). Slides were viewed, and images were captured with a confocal microscope (LSM780, Zeiss, Jena, Germany).

### Fluoro-Jade C Staining

Fluoro-Jade C (F-JC) is a polyanionic fluorescein derivative that binds to degenerating neurons with high sensitivity and specificity. F-JC staining was performed to detect the neuronal damage around the hematoma 24 h post-ICH using the standard protocol described by Ehara and Ueda (2009). Briefly, after drying in hot air for 2 h, selected sections were incubated with a solution of 1% NaOH in 80% ethanol for 5 min and then rehydrated in 70% ethanol and distilled water for 2 min each. Slices were then incubated with 0.06% KMnO_4_ for 10 min, rinsed with distilled water for 2 min, and incubated with a 0.0001% solution of F-JC (Millipore, Temecula, CA, United States) for 20 min. Slices were washed with distilled water three times for 1 min each. Sections were observed and photographed under a fluorescence microscope (Olympus OX51, Tokyo, Japan). The number of F-JC-positive neurons was counted and calculated using Image-Pro Plus v6.0 image analysis software (Media Cybernetics, Rockville, MD, United States).

### TUNEL Staining

Brain samples were collected 24 h after ICH. Each group of samples was fixed with a formalin solution and dehydrated with paraffin to preserve the cell structure; the paraffin block was then sliced into 3-μm thick sections. Cell death around the hematoma was detected using the *In Situ* Cell Death Detection Kit (Roche, Mannheim, Germany) according to the manufacturer’s instructions, followed by staining with 3, 3′-diaminobenzidine for illustration. Each section was observed and photographed under a Zeiss Axio Imager A2 light microscope. The number of TUNEL-positive cells was counted and calculated using Image-Pro Plus v6.0 image analysis software (Media Cybernetics, Rockville, MD, United States).

### Evans Blue Extravasation and Fluorescence

Blood–brain barrier disruption was evaluated using Evans blue dye extravasation, as described in our previous study (Chen et al., 2015). Briefly, at 24 h post-operation, Evans blue dye (2%, 5 ml/kg; Sigma-Aldrich, St. Louis, MO, United States) was injected into the left femoral vein of anesthetized rats over a 2-min period and allowed to circulate for 60 min. Rats were sacrificed by an intracardial perfusion with sterile saline. Brain samples were then weighed, homogenized in saline, and centrifuged at 15,000 *g* for 30 min. Next, an equal volume of trichloroacetic acid was added to the resulting supernatant. Samples were then incubated overnight at 4°C and centrifuged again at 15,000 *g* for 30 min. The resulting supernatant was then spectrophotometrically quantified at 620 nm to determine the amount of extravasated Evans blue dye.

For Evans blue fluorescence, sterile saline was replaced with 4% paraformaldehyde after intracardial perfusion. Then, brain specimens were removed, and coronal brain sections (10 μm) were prepared using a procedure similar to the method described for immunohistochemistry. The red auto-florescence of the Evans blue dye was then observed in sections using excitation and emission filters for red fluorescence (Olympus OX51, Tokyo, Japan).

### Magnetic Resonance Imaging

T2^∗^-weighted imaging was performed using previously described methods to evaluate the hematoma sizes in the ICH + vehicle and ICH + 150-pmol HC-067047 groups 24 h after autologous-blood-induced ICH (Wang et al., 2018). MRI scans were performed in a 7.0-T Varian MR scanner (Bruker, United States). Rats were anesthetized with 2% isoflurane in ambient air during each imaging procedure. Contiguous coronal slices of the hematoma were imaged with a resolution matrix = 256 × 256, a field of view (FOV) = 35 mm × 35 mm, a slice number = 20, and a slice thickness = 1 mm. Hematoma volumes were calculated as previously described (Okauchi et al., 2009; Dang et al., 2017). All the images were analyzed using ImageJ software (National Institutes of Health, Bethesda, MD, United States), and volume measurements were performed by two observers in a blinded manner.

### Western Blotting

Protein samples were extracted from brain tissues around the lesion sites using a protein extraction kit (Beyotime Biotechnology, Co., Ltd., Jiangsu, China) supplemented with a protease inhibitor cocktail (Roche, Indianapolis, IN, United States). Equal amounts of extracted proteins were separately loaded on SDS–polyacrylamide gels and subjected to immunoblotting. The following primary antibodies were used: rabbit polyclonal anti-TRPV4 antibody (1:500, Alomone Labs, Jerusalem, Israel); mouse monoclonal anti-VE-cadherin antibody (1:200, Santa Cruz Biotechnology, Santa Cruz, CA, United States); rabbit polyclonal anti-Occludin antibody (1:500, Invitrogen, Grand Island, NY, United States); mouse monoclonal anti-Claudin-5 antibody (1:500, Invitrogen, Grand Island, NY, United States); rabbit polyclonal anti-p-PKCα antibody (1:500, Cell Signaling Technology, Boston, MA, United States); and rabbit polyclonal anti-p-MLC2 antibody (1:500, Cell Signaling Technology, Boston, MA, United States). A β-actin antibody (1:1000, Beyotime, Shanghai, China) was used as an internal loading control. Appropriate secondary antibodies (Santa Cruz Biotechnology, Santa Cruz, CA, United States) were incubated with the membranes for 1 h at room temperature. The immunoreactive bands were visualized using enhanced chemiluminescence (Amersham Biosciences, Arlington Heights, IL, United States) according to the manufacturer’s instructions and visualized with an imaging system (VersaDoc MP 4000; Bio-Rad, Hercules, CA, United States). Data were analyzed using ImageJ software (National Institutes of Health, Bethesda, MD, United States).

### Evaluation of RhoA Activity

RhoA activity was measured using a commercial kit (Cytoskeleton Inc., Denver, CO, United States) (Manaenko et al., 2016). Two equal aliquots of each sample were collected. One aliquot was processed according to the manufacturer’s recommendations and used to evaluate RhoA activity. The second aliquot was probed with an antibody against β-actin as the loading control.

### F-Actin/G-Actin Assay

Actin polymerization was evaluated using the G-actin/F-actin *In Vivo* Assay Biochem Kit (Cytoskeleton, Inc.), according to the manufacturer’s instructions (Shi et al., 2017). Briefly, lysates were prepared from brain tissues around the lesion sites using the Lysis and F-actin Stabilization Buffer provided in the kit. F-actin and G-actin were separated by centrifugation at 100,000 × *g* for 1 h at 37°C. The supernatant containing G-actin was collected, and the F-actin in the pellets was depolymerized to the globular form using F-actin Depolymerization Buffer. Samples from both the G- and F-actin components were examined by western blotting. The membrane was stripped several times and then re-probed with anti-actin antibodies as described above.

### Statistical Analysis

Data are expressed as the means ± standard errors of the means. Neurobehavioral data were analyzed using the Kruskal–Wallis one-way analysis of variance on ranks followed by the Student–Newman–Keuls test. All other data were analyzed using one-way analysis of variance followed by the Tukey *post hoc* test. *P*-values < 0.05 were considered statistically significant. All statistical analyses were performed using SigmaPlot 10.0 for Windows (Systat Software Inc., San Jose, CA, United States).

## Author Contributions

HZ, HF, and YC designed the experimental protocols. HZ, KZ, RT, HM, YZ, and PW performed the experiments. YC, RH, and XL carried out the data analysis. HZ and YC prepared and revised the manuscript. YC and HF gave the final approval of manuscript to be published.

## Conflict of Interest Statement

The authors declare that the research was conducted in the absence of any commercial or financial relationships that could be construed as a potential conflict of interest.

## Supplementary Material

FIGURE S1 The effects of HC-067047 administration on hematoma volume after ICH. **(A)** Representative T2^∗^-weighted images and **(B)** quantitative analyses of hematoma size in the vehicle and HC-067047 treatment groups (150 pmol per rat) 24 h after the operation are shown, *n* = 5 rats per group. *p* > 0.05 compared with the vehicle group.

FIGURE S2 The effects of H7 or C3 transferase administration on activation of the RhoA/MLC2 pathway and BBB integrity after ICH. **(A)** Representative bands and quantitative analysis of RhoA activation and MLC2 phosphorylation in the sham, vehicle, and H7 groups 24 h after the operation are shown, *n* = 5 rats per group. **(B)** Representative bands and quantitative analysis of MLC2 phosphorylation in the sham, vehicle, and C3 transferase groups 24 h after the operation are shown, *n* = 5 rats per group. **(C)** Results of the Evans blue extravasation evaluation performed 24 h after the operation in the sham, vehicle, H7, and C3 transferase groups, *n* = 6 rats per group. Data are presented as the means ± standard errors of the means. ^∗∗^*p* < 0.01 compared with the sham group; ^#^*p* < 0.05 compared with the vehicle group.

## Abbreviations

AA: arachidonic acid
BBB: blood–brain barrier
Ca^2+^: calcium
F-actin: filamentous actin
G-actin: globular actin
ICH: intracerebral hemorrhage
MCAO: middle cerebral artery occlusion
MMPs: matrix metalloproteinases
TRP: transient receptor potential
TRPV4: transient receptor potential vanilloid 4
ZO: zonula occludens
