# Supplementary figures and images for: TRPV4 Blockade Preserves the Blood–Brain Barrier by Inhibiting Stress Fiber Formation in a Rat Model of Intracerebral Hemorrhage
Source: Front Mol Neurosci. 2018 Mar 27;11:97. doi: 10.3389/fnmol.2018.00097 (PMC5880899; doi:10.3389/fnmol.2018.00097)

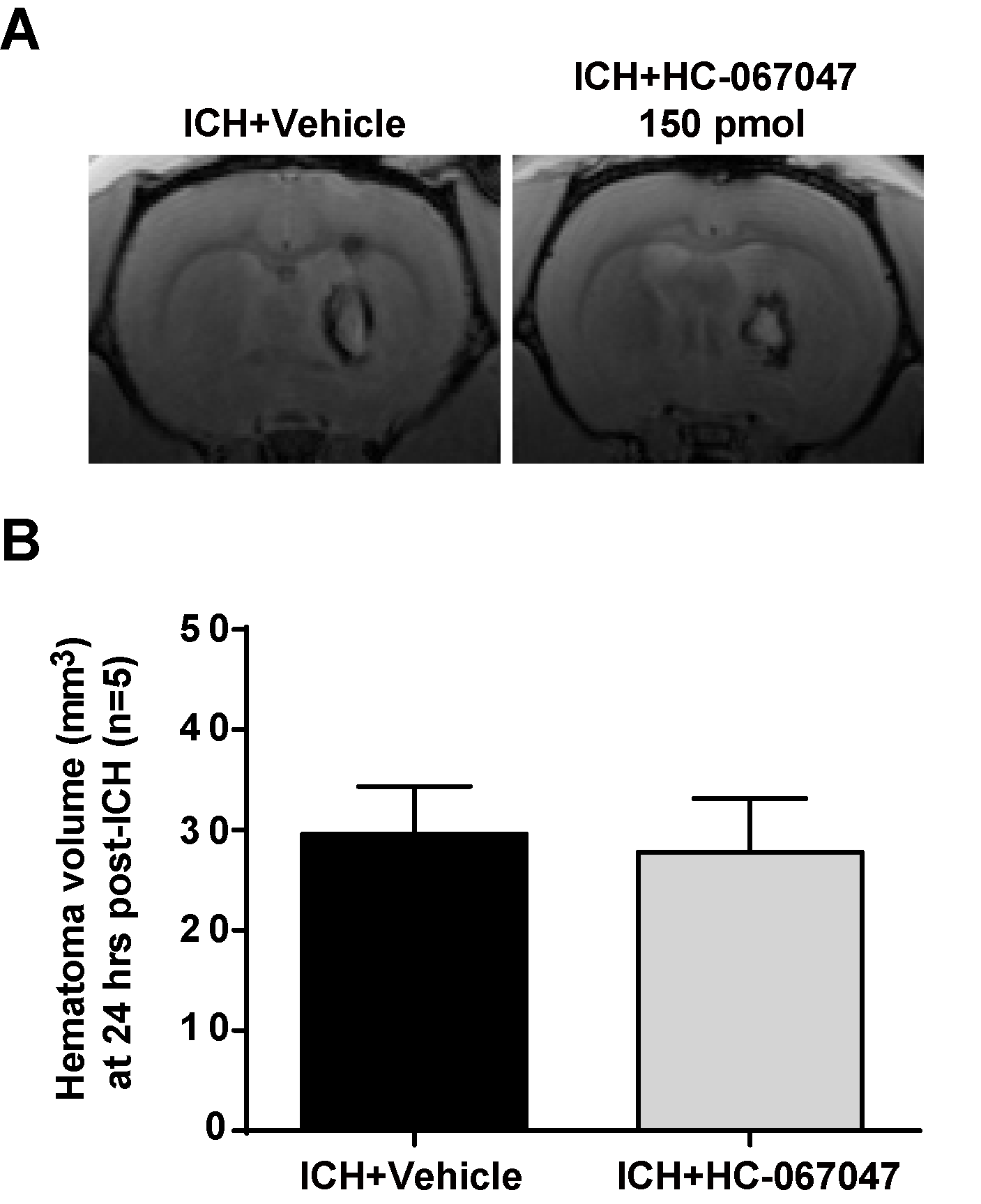

Supplement: FIGURE S1 — The effects of HC-067047 administration on hematoma volume after ICH. (A) Representative T2∗-weighted images and (B) quantitative analyses of hematoma size in the vehicle and HC-067047 treatment groups (150 pmol per rat) 24 h after the operation are shown, n = 5 rats per group. p > 0.05 compared with the vehicle group. [file Image_1.TIF]

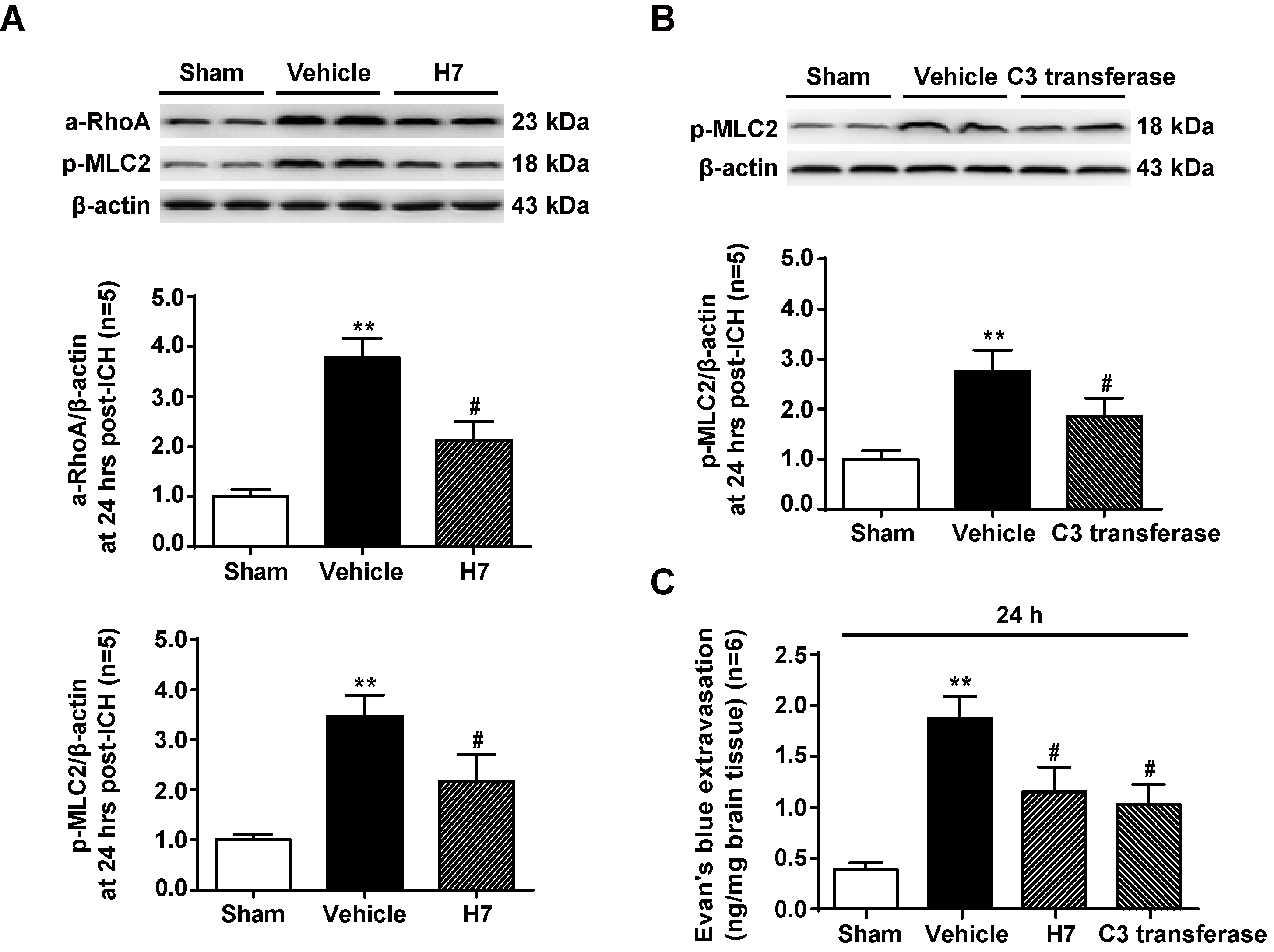

Supplement: FIGURE S2 — The effects of H7 or C3 transferase administration on activation of the RhoA/MLC2 pathway and BBB integrity after ICH. (A) Representative bands and quantitative analysis of RhoA activation and MLC2 phosphorylation in the sham, vehicle, and H7 groups 24 h after the operation are shown, n = 5 rats per group. (B) Representative bands and quantitative analysis of MLC2 phosphorylation in the sham, vehicle, and C3 transferase groups 24 h after the operation are shown, n = 5 rats per group. (C) Results of the Evans blue extravasation evaluation performed 24 h after the operation in the sham, vehicle, H7, and C3 transferase groups, n = 6 rats per group. Data are presented as the means ± standard errors of the means. ∗∗p < 0.01 compared with the sham group; #p < 0.05 compared with the vehicle group. [file Image_2.TIF]
